# Comparison of Immunohistochemical Expression of Antiapoptotic Protein Survivin in Normal Oral Mucosa, Oral Leukoplakia, and Oral Squamous Cell Carcinoma

**DOI:** 10.1155/2015/840739

**Published:** 2015-09-20

**Authors:** Amita Negi, Abhiney Puri, Rakhi Gupta, Rajat Nangia, Alisha Sachdeva, Megha Mittal

**Affiliations:** ^1^Department of Oral Pathology & Microbiology, Himachal Dental College, Sunder Nagar, District Mandi, Himachal Pradesh 175002, India; ^2^Department of Oral Pathology & Microbiology, Himachal Institute of Dental Sciences, Paonta Sahib, District Sirmour, Himachal Pradesh 173025, India

## Abstract

*Background*. Oral squamous cell carcinoma is the sixth most frequent malignant tumor worldwide and the third most common cancers in developing countries. Oral leukoplakia is the best-known precursor lesion of oral squamous cell carcinoma. The aim of the present study was to compare immunohistochemical expression of antiapoptotic protein survivin in normal oral mucosa, oral leukoplakia, and oral squamous cell carcinoma. *Method*. Total 45 specimens of formalin fixed paraffin embedded tissue blocks, 15 in each of the following: normal oral mucosa, leukoplakia, and oral squamous cell carcinoma were used for the study. Immunohistochemical reaction for survivin protein was performed for the 4 *µ*m thick histological sections taken on positively charged slides. *Results*. 20% normal mucosa cases, 53.33% cases of leukoplakia, and 80% of oral squamous cell carcinoma were found out to be survivin positive. One way ANOVA test indicated statistically significant difference of survivin expression between the three different groups (*p* < 0.001). *Conclusion*. A high incidence of survivin protein expression in oral epithelial dysplasia and squamous cell carcinoma samples indicate that survivin protein expression may be an early event in initiation and progression of oral squamous cell carcinoma.

## 1. Introduction 

Carcinoma in the oral region is the sixth most frequent malignant tumor throughout the world and the third commonly occurring cancer in developing countries, with especially high incidence in South-East Asian countries and India [1, 2]. Oral cavity is amongst one of the five major locations of cancer in India, representing 19% of the total count of cancer in males and 7% cases of cancer in females. Approximately 90% of oral malignancies are squamous cell carcinomas (SCC), arising in the mucosal lining. The predominance of oral carcinoma is because of extensive consumption of tobacco and its products in India [3].

In the oral mucosa, almost all carcinomas are preceded by occurrence of a precancerous lesion, progressing into cancer at a later stage. Oral leukoplakia is the most well recognized forerunner of oral cancer. Risk of malignant transformation is variable but many studies have reported that the progression of precursor lesions into cancer is between <1 and 18% [4].

Oral cancer develops as a result of increased lack of genetic stability that involves stimulation of oncogenes and switching off the tumor suppressor genes. In their early stages, most of the human cancers are characterized by loss of cellular systems that regulate cell cycle progress, cell death versus growth balance, and apoptosis [5].

It is now well established that, in a tumor cell, active inhibition of apoptosis occurs due to unchecked division along with numerous genetic modifications. The molecular mechanisms in the progression of apoptosis as well as its regulating factors are extremely conserved in evolution. In recent years, a unique gene known as survivin that encodes for a structurally distinctive suppressor of apoptosis has been recognized [5].

*Survivin* is a 16.5 kDa intracellular protein from the inhibitor of apoptosis (IAP) gene family. All metazoans genomes contain IAP molecules that characteristically consist of 1–3 copies of ~70-amino-acid zinc-finger fold, known as the baculovirus IAP repeat (BIR). These proteins get physically associated with initiator and effector caspases, intercepting their proteolytic maturation and enzyme activity and hence inhibiting apoptosis [6].

The survivin gene, locus 17q25, is expressed in the G2/M phase of the cell cycle and inhibits apoptosis following physical association with the mitotic spindle. It is now evident that survivin occurs in two immunohistochemically different pools. The nuclear pool is circumscribed to kinetochores of metaphase chromosomes and to the central spindle midzone at anaphase, and the cytosolic pool integrated with interphase microtubules, centrosomes, spindle poles, and mitotic spindle microtubules at metaphase and anaphase [7–9].

Therefore, in the present study, we intended to evaluate the immunohistochemical expression of IAP protein survivin in formalin fixed paraffin embedded (FFPE) sections of normal oral mucosa, oral leukoplakia, and oral squamous cell carcinoma (OSCC) and to find out the correlation between the expressions of survivin within the three groups.

## 2. Material and Methods

The sample for the study consisted of total 45 specimens of FFPE tissue blocks, 15 in each of the following: normal oral mucosa, leukoplakia, and OSCC. The leukoplakia cases were histopathologically categorized as mild dysplasia (5 cases), moderate dysplasia (9 cases), and severe dysplasia (1 case). The cases of the OSCC group were histopathologically categorized as histopathologically categorized as well differentiated SCC (4 cases), moderately differentiated SCC (9 cases), and poorly differentiated SCC (2 cases). Immunohistochemical reaction for survivin protein was performed for the 4 *μ*m thick histological sections taken on* positively charged slides*. Antigen retrieval was performed by heat induced epitope retrieval (HIER) technique using microwave (EZ retriever system, BioGenex). For this, sections were immersed in 10 mM sodium citrate buffer (pH6.0) in the containers supplied with the retrieval system and two cycles were run: first cycle at 85°C for 10 minutes and second cycle at 100°C for 15 minutes. Anti-survivin rabbit monoclonal antibody (BioGenex, Fremont CA, USA) and Super Sensitive Polymer Detection System (BioGenex, Fremont CA, USA) were used for detection of survivin. The high grade human breast carcinoma specimen showing strong expression for survivin was used as a positive control. For negative control, primary antibody was replaced with PBS.

Survivin positivity was detected in each section. 1000 cells per section were counted under 400x magnification and percentage of survivin positive cells was calculated. The percentage of positive cells was scored according to the method of Nakagawa et al. [10] as follows:

 3+ = strong staining (more than 50% stained).
 2+ = moderate staining (between 25 and 50% stained).
 1+ = weak staining (between 5 and 25% stained).
 0 = negative (less than 5% stained).

*One way ANOVA* (analysis of variance) test was done to test the statistical significance of the difference in the number of survivin positive cells between the three groups.

## 3. Results

**Figure 1:**
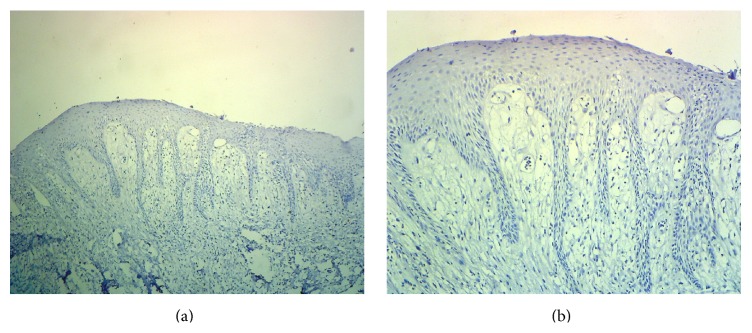
Representative photomicrographs of the immunohistochemical staining for survivin in normal oral mucosa at (a) 40x and (b) 100x magnification showing negative staining.

**Figure 2:**
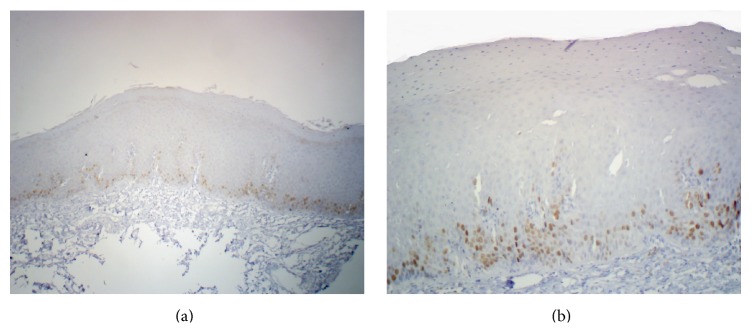
Representative photomicrographs of the immunohistochemical staining for survivin in a case of oral leukoplakia (a) 40x and (b) 100x magnification showing positive nuclear staining.

**Figure 3:**
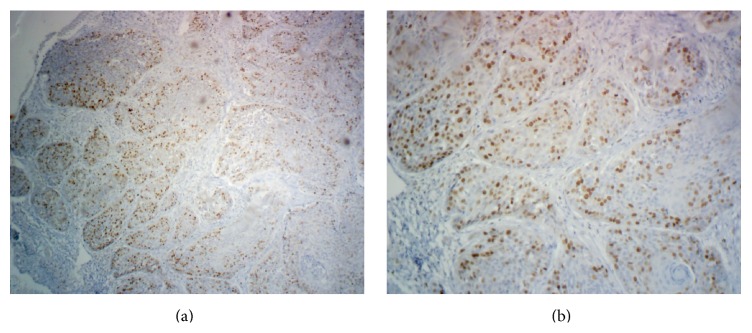
Representative photomicrographs of the immunohistochemical staining for survivin in a case of oral squamous cell carcinoma at (a) 40x and (b) 100x magnification showing positive nuclear staining.

**Figure 4:**
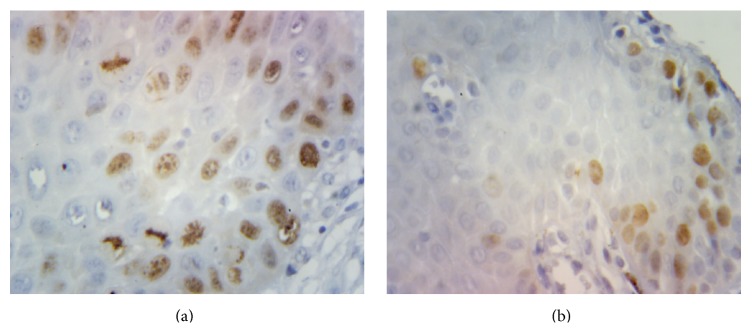
Representative photomicrographs of the immunohistochemical staining for survivin in a case of (a) oral squamous cell carcinoma and (b) oral leukoplakia at 400x magnification showing positive nuclear staining.

Three out of fifteen normal mucosa cases (20%) showed survivin positivity and the rest were all negative (Figures 1(a) and 1(b)). All the positive cases were having number of positive cells less than 5% and hence all were given a score of 0. Eight out of fifteen (53.33%) cases of leukoplakia were found to be survivin positive (Figures 2(a), 2(b), and 4(b)). Total 13 (86.67%) cases were having a score of 0 (total survivin positive cells count less than 5%) and two cases (13.33%) were given a score of 1+ (total survivin positive cells count between 5 and 25%). Twelve out of fifteen cases (80%) of OSCC were found out to be survivin positive (Figures 3(a), 3(b), and 4(a)). Total four cases (26.67%) were given a score of 0, six cases (40%) were given a score of 1+, and five cases (33.33%) were given a score of 2+.

The statistical analysis (one way ANOVA, Table 1) indicated that the difference of survivin positive cells/1000 cells observed between the three different groups, that is, normal oral mucosa, leukoplakia, and OSCC, was statistically significant (*p* < 0.001).

Furthermore,* Bonferroni test* (Table 2) was applied to evaluate each comparison (i.e., normal oral mucosa with leukoplakia and OSCC, leukoplakia with normal oral mucosa and OSCC, and OSCC with normal oral mucosa and leukoplakia) of the significance test. This analysis showed that the difference in number of survivin positive cells was statistically insignificant between normal oral mucosa and leukoplakia, whereas it was found to be statistically significant between leukoplakia and OSCC (*p* < 0.001). Also, the difference between normal oral mucosa and OSCC was found to be significant (*p* < 0.001).

## 4. Discussion

Oral carcinoma is the most prevalent cancer in the head and neck region with unfavourable outcome. In spite of the advancements in the treatment protocols like radiation therapy, advanced surgical procedures, and the emergence of aggressive chemotherapy regimens, the 5-year survival rate of OSCC is only 35–50% [11]. One of the major causes for the unfavourable prognosis in oral cancer is the inability to detect the disease at an early stage and its progression. Therefore, a strong need arises for distinct and specific molecular tumor markers to predict the tumor progression and prognosis. Although plenty of tumor markers and predisposing factors are well recognized for oral carcinoma, their role in determining the prognosis is still not established [12].

A tumor establishes itself whenever there are compromises in the regulation of cell proliferation as well as in the control of cell death. In this direction, many gene products are known to be potentially responsible for modifications in tumor cell viability, the resistance to apoptosis, and the magnification of tumor progression. IAP proteins are now known as modulators of downstream events in the progression of apoptosis directly involving the inhibition of terminal effector caspases 3 and 7 and ultimately hampering apoptosis [13].

Survivin has been initially seen to be expressed in fetal life and not in differentiated adult tissue. However, further studies showed positive expression of survivin in normal endometrium, thymus, and placenta, all of them representing tissues with higher proliferation capacity [14]. Survivin has also been reported to be expressed in several human cancers including cancers of lung, breast, bladder, gastrointestinal tract, and haematological tumors [7, 13, 15].

Survivin is the one representative of the IAP group of proteins which has been recognized to have a role in the nucleus; hence it could be presumed that normal epithelium is dedifferentiated while development and progression of carcinoma result in reexpression of survivin [11]. Therefore survivin expression can be helpful for evaluating the progress of head and neck carcinomas.

In the present study, we calculated 1000 cells per section showing positive survivin expression and the results were compared for the test of significance using one way ANOVA test. We found a statistically significant difference between the three groups (*p* < 0.001).

Further Bonferroni analysis was performed to do the multiple comparisons. This test showed that the difference in number of survivin positive cells and their percentage was statistically insignificant between normal oral mucosa and leukoplakia. These results were in accordance with study done by Lo Muzio et al. [16].

In our study, survivin positivity in normal oral mucosa was observed sporadically in basal layer only and all the positive cases were scored as 0; that is, the number of positive cells/1000 cells was between 0 and 5%. This was in accordance with the studies done by Lo Muzio et al. [17].

Further Bonferroni analysis was performed to do the multiple comparisons. This test showed that the difference in number of survivin positive cells and their percentage was statistically insignificant between normal oral mucosa and leukoplakia. These results were similar to the study done by Lo Muzio et al. [17]. In contrast to these results, the difference was significant in studies by Khan et al. [5] and Tanaka et al. [18]. This could be attributed to comparatively small sample size in our study.

The difference in number of survivin positive cells was statistically significant (*p* < 0.001) between normal oral mucosa and OSCC and between leukoplakia and OSCC. These findings are similar to study conducted by Pannone et al. [19].

We observed an upregulation of survivin expression in leukoplakia and OSCC. More than half of the cases of leukoplakia in our study showed positive survivin expression. Thus, we can postulate that, during the development of oral cancer, antiapoptotic protein survivin might accumulate within the involved tissue at an early stage. Numerous types of precancerous lesions or initial stages of cancer have been reported to show positive expression of survivin, including colon polyps and Bowen disease [20] and precancerous colorectal lesion [21].

Upregulation of survivin in OSCC and highly significant difference with leukoplakia were observed in our study. However, we did not observe any significant relation between degree of survivin expression and the histological grade of OSCC. Along with small sample size, the nonuniform proportion of various grades of dysplasia and histological grades of OSCC could be the reason for not significant difference in survivin expression within the groups.

Hence, in conclusion, our study demonstrated a high expression of survivin protein in leukoplakia and SCC cases. This expression may occur as an early phenomenon in the beginning and advancement of OSCC. Also, survivin may act as an important therapeutic target because of its unique expression in tumor cells and its absence in most adult tissues.

Although our study demonstrated the positive immune-expression of survivin as a reliable marker for OSCC, further studies are required with larger number of samples to assess the relation of survivin with different histological grades of squamous cell carcinoma. Also, to confirm the role of survivin as a guide to prognosis of OSCC, studies with clinical follow-up are desired.

## Figures and Tables

**Table 1:** One way ANOVA analysis between the three groups showing statistically significant difference.

	Sum of squares	df	Mean square	*F*	*p* value
Number of positive cells/1000 cells					
Between groups	237224.711	2	118612.356	16.547	<0.001^*∗*^
Within groups	301064.933	42	7168.213		
Total	538289.644	44			

^*∗*^ *p* value statistically significant.

**Table 2:** Bonferroni analysis showing multiple comparison.

Dependent variable			Std. error	*p* value
Number of positive cells/1000 cells	Normal mucosa	Leukoplakia	30.91540	1.000
		Squamous cell carcinoma	30.91540	<0.001^*∗*^
	Leukoplakia	Normal mucosa	30.91540	1.000
		Squamous cell carcinoma	30.91540	<0.001^*∗*^
	Squamous cell carcinoma	Normal mucosa	30.91540	<0.001^*∗*^
		Leukoplakia	30.91540	<0.001^*∗*^

^*∗*^ *p* value statistically significant.
